# A phase I study of HEC73543, an oral FLT3 inhibitor: the effect of food on pharmacokinetics after oral dosing in healthy Chinese volunteers

**DOI:** 10.3389/fphar.2025.1636504

**Published:** 2025-10-08

**Authors:** Di Wang, Min Wu, Xiaojiao Li, Yingzhi Jiang, Bing Liu, YingJun Zhang, Li Deng, Yanhua Ding

**Affiliations:** ^1^ Department of Phase I Clinical Trial Unit, The First Hospital of Jilin University, Changchun, Jilin, China; ^2^ Sunshine Lake Pharma Company, Ltd., Dongguan, China; ^3^ State Key Laboratory of Anti-Infective Drug Discovery and Development, Sunshine Lake Pharma Company, Ltd., Dongguan, China

**Keywords:** AML, HEC73543, FLT3 inhibitor, food effect, pharmacokinetics

## Abstract

**Objective:**

This study evaluated the effect of food on the pharmacokinetics (PK) and safety of HEC73543.

**Methods:**

This randomized, open-label, single-dose, phase I parallel trial included 40 healthy subjects randomized (1:1) to either high-fat or fasted groups. Participants received a single oral dose of 40 mg HEC73543. Blood samples were collected and detected using a validated liquid chromatography tandem mass spectrometry method. PK parameters were calculated using non-compartmental methods. Safety was monitored throughout the study.

**Results:**

For the HEC73543, the fed-to-fasted ratios were: area under the curve from time 0 to time t (AUC_0–t_), 219.42% (90% confidence interval [CI]: 173.76, 277.08%); AUC from zero to infinity ( ${\text{AUC}}_{0–\infty }$ ), 255.22% (90% CI: 198.10, 328.80%); and maximum concentration (C_max_), 221.31% (90%CI: 190.57, 257.00%). Similarly, for the metabolite M3, the fed-to-fasted ratios were: AUC_0–t_, 190.86% (90%CI: 153.26%, 237.68%); ${\text{AUC}}_{0–\infty }$ , 190.29% (90% CI: 151.77%, 238.59%); and C_max_, 177.48% (90% CI: 137.80%, 228.58%). Median T_max_ of HEC73543 were comparable between the two groups. The most frequently Treatment-Related Adverse Events (TRAEs) were elevated blood triglycerides, oral ulceration, hyperuricemia, diarrhea, thoracalgia. Most TRAEs were Grade 1 or 2.

**Conclusion:**

High-fat food intake enhanced bioavailability and increases the systemic exposure levels of HEC73543 and its metabolite M3.

**Clinical Trial Registration:**

NCT05454098 (http://www.clinicaltrials.gov/).

## 1 Introduction

Acute myeloid leukemia (AML) accounts for the majority of acute leukemia cases in adults and remains a major clinical challenge (Bhansali et al., 2023), and is associated with a poor prognosis, with a 5-year survival rate of only about 30%–35% (Kantarjian et al., 2021). Among the molecular alterations in AML, FLT3 mutations are common, occurring in approximately 30% of newly diagnosed cases (Abdel-Aziz et al., 2023). The two primary types of FLT3 mutations are internal tandem duplication (ITD) mutations and tyrosine kinase domain (TKD) mutations, with reported frequencies of 30% (Vela et al., 2020; Helbig et al., 2020) and 7%–10% (Fedorov et al., 2023), respectively. FLT3-ITD is an important prognostic biomarker (Daver et al., 2019). As indicated by the 2022 European LeukemiaNet (ELN) risk stratification, patients with FLT3-ITD-positive AML are categorized as an intermediate-risk subtype (D€ Ohner et al., 2022). Targeted inhibition of FLT3 has emerged as a promising strategy in precision medicine. FLT3 inhibitors have been developed to improve treatment outcomes in high-risk AML patients, with Gilteritinib, a second-generation type I FLT3 inhibitor, being the FDA-approved monotherapy for relapsed/refractory (R/R) AML harboring FLT3 mutations (Perl, 2019). It is the first FLT3 inhibitor to be approved for monotherapy, and *ex vivo* studies have demonstrated that gilteritinib markedly suppresses the colony-forming capacity of FLT3-ITD–positive leukemia samples, especially in those with a high allelic ratio (AR ≥ 0.5) (Cucchi et al., 2018). Meanwhile, multiple novel agents are currently undergoing clinical development to further improve treatment efficacy and safety.

HEC73543 is a second generation, highly specific, orally administered FLT3 inhibitor, innovatively developed by Dongguan HEC Biopharmaceutical R&D Co. (Liu et al., 2025), with global intellectual property rights. It is designed for FLT3-ITD mutation AML patients, either as monotherapy or in combination with other therapeutic agents. HEC73543 is an oral tablet. It is a weak base (pKa 8.02) with high lipophilicity (logP 4.98) and exhibits very low aqueous solubility, food might affect its rate and extent of absorption by altering the luminal conditions in the human gastrointestinal tract (Budha et al., 2012). Therefore, a food-effect study is necessary to evaluate the potential impact of fed conditions on the systemic exposure of HEC73543. Before the present study, a single-agent Phase I clinical pharmacodynamic trial is ongoing, but results remain incomplete. After oral administration of HEC73543, 15 metabolites have been detected in human plasma, with the parent drug and the metabolite M3 being predominant. Urine and feces have not yet been analyzed. Nonclinical pharmacokinetic findings suggest that HEC73543 can be metabolized via CYP3A4. To our knowledge, this is the first report on the pharmacokinetics of HEC73543 in humans. We believe that publishing these data will contribute important foundational knowledge to support the ongoing clinical development of HEC73543 and provide useful references for researchers and clinicians working on FLT3-targeted therapies.

## 2 Materials and methods

### 2.1 Ethical approval

This study protocol was reviewed and approved by the Ethics Committee of the Clinical Research Institute, the First Hospital of Jilin University (Changchun, China). The ethics approval number is 22Y089-002. All study procedures were conducted in accordance with the standards of the Phase I Clinical Trial Unit of the First Hospital of Jilin University. Furthermore, the study was conducted in full compliance with the Declaration of Helsinki and the Guidelines for Good Clinical Practice (GCP). Written informed consent was obtained from all participants before their enrollment in the study.

### 2.2 Subjects

Healthy volunteers aged 18–45 years were enrolled in this trial. The eligibility criteria included a body weight of ≥45 kg for females and ≥50 kg for males, with a body mass index (BMI) between 18 and 28 kg/m^2^. The primary exclusion criteria were as follows: 1) a known history of cardiovascular, central nervous system, hepatic, renal, or other systemic diseases; 2) abnormal findings on a 12-lead electrocardiogram (ECG) during screening; 3) smoking more than five cigarettes per day; 4) a history of severe drug or food allergies; 5) use of drugs that are substrates of P-glycoprotein (P-gp) or breast cancer resistance protein (BCRP), or inhibitors/inducers of CYP3A4 within 14 days before screening; 6) consumption of caffeine-containing beverages or foods within 48 h before trial initiation; and 7) intake of alcohol or alcohol-containing beverages within 24 h before drug administration; 8) inability to tolerate a standardized meal; 9) positive test results for hepatitis B surface antigen (HBsAg), hepatitis C virus (HCV) antibody, human immunodeficiency virus (HIV) antibody, or Treponema pallidum (syphilis) antibody. And the acceptable reference ranges for clinical hematology and biochemistry assessments are detailed in Table 2.

### 2.3 Study design and administration

**FIGURE 1:**
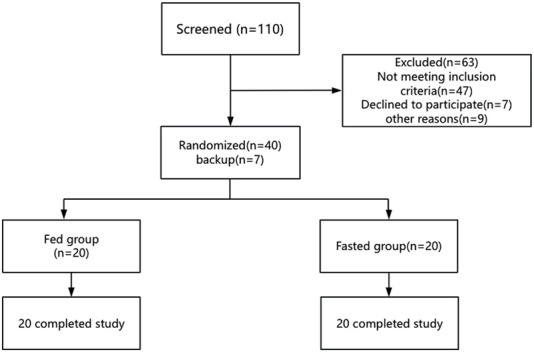
Study design flowchart.

This was a phase I, randomized, open-label, parallel-design trial conducted in healthy volunteers (ClinicalTrials.gov NCT05454098). The trial employed a stratified block randomization method, with stratification factors including gender (male vs. female) and BMI (18 ≤ BMI < 24 vs. 24 ≤ BMI ≤28). Based on the Guideline on the Investigation of Bioequivalence of European Medicines Agency, which recommend enrolling at least 12 subjects per dietary condition group (European Medicines Agency, Committee for Medicinal Products for Human Use (CHMP) (2010)), a total of 40 healthy volunteers were randomly assigned in a 1:1 ratio to either the fed group (receiving a high-calorie meal of approximately 800–1,000 kcal (U.S. Food and Drug Administration, 2022) 30 min before drug administration) or the fasted group (fasting for at least 10 h prior to drug administration). The flowchart of the study design is shown in Figure 1. A mouth check was conducted to confirm that the drug was taken. All participants completed the drug administration and were included in the subsequent analysis.

### 2.4 Safety assessment

TEAEs were classified according to the Common Terminology Criteria for Adverse Events (CTCAE) version 5.0, as established by the National Cancer Institute. TEAEs were closely monitored throughout the study to evaluate their severity, frequency, and causal relationship with the drug. Safety was evaluated based on vital signs, including body temperature, heart rate, and blood pressure, as well as physical examinations, 12-lead ECGs, and clinical laboratory tests. Laboratory tests included hematology, serum biochemistry, urinalysis, and coagulation parameters.

### 2.5 PK assessment

For PK evaluation, 4 mL blood samples were collected into EDTA-K_2_ tubes at the following time points post-dose: 0, 1, 2, 3, 4, 5, 6, 8, 12, 24, 48, 72, 96, 120, 168, 216, 264, 312, 360, 432, 504, 576, and 648 h. The samples were centrifuged at 2,600 g for 10 min, then all plasma samples were stored at −60 °C or below. According to the validated bioanalytical method, both HEC73543 and its metabolite M3 were demonstrated to be stable in human plasma under these storage conditions for at least 244 days. In this trial, the actual maximum sample storage duration (from sample collection to the completion of analysis) was 69 days, well within the validated stability period. The plasma concentrations of HEC73543 and its metabolite M3 were determined using a validated LC-MS/MS method developed and fully validated by Suzhou Guochen Bio-Technology Co., Ltd. the method uses HEC82652 and HEC82781 as internal standards for HEC73543 and M3, respectively. After protein precipitation, plasma samples were analyzed with a quantification range of 8.00–12,000 ng/mL for HEC73543 and 4.00–6,000 ng/mL for M3. Incurred sample reanalysis (ISR) was performed on 120 plasma samples (≥10% of total samples). The results showed that 96.7% of samples had differences between −15.1% and 14.5%, meeting the acceptance criterion of at least two-thirds of samples within ±20%. The plasma was then analyzed to calculate the PK parameters using non-compartmental analysis (NCA), including the T_max_, C_max_, AUC_0-t_ and ${\text{AUC}}_{0–\infty }$ , mean retention time (MRT), and terminal elimination half-life (t_1/2_). Because only oral dosing was evaluated, absolute bioavailability (F) is unknown; therefore, true clearance (CL) and volume of distribution (V) cannot be estimated; only the apparent parameters CL/F and Vz/F are reported.

### 2.6 Statistical analysis

Descriptive statistics were presented as mean with standard deviation (SD), geometric mean with geometric coefficient of variation (GCV), or median with range (minimum–maximum), as appropriate. Statistical analyses were performed using SAS software (version 9.4; SAS Institute Inc., Cary, NC, United States).

Primary PK parameters, including C_max_, AUC_0-t_, and ${\text{AUC}}_{0–\infty }$ , were analyzed using Phoenix WinNonlin software (version 8.3, Pharsight Certara Company). Natural logarithms of these parameters were calculated for the fed and fasted groups, with the fasted group serving as the reference. The average bioequivalence (ABE) analysis was conducted to calculate the geometric mean ratios (GMRs) and their 90% CIs for HEC73543 and M3 between the fed and the fasted groups.

## 3 Results

### 3.1 Demographics and baseline of the subjects

A total of 40 healthy Chinese subjects were enrolled and dosed in this study. The demographic characteristics of the fasted and fed groups are summarized in Table 1. Baseline clinical hematology and biochemistry parameters were comparable between the two groups, with no statistically significant differences observed (*p* > 0.05), as shown in Table 2.

**TABLE 1:** Demographic characteristics and baseline of two groups.

Characteristics (Units)	Fasted (n = 20)	Fed (n = 20)	Total (n = 40)
Age (y), mean (SD)	34.8 (6.9)	32.3 (7.4)	33.5 (7.2)
Male, n (%)	10 (50)	9 (45)	19 (47.5)
Body weight (kg), mean (SD)	65.2 (8.1)	63.88 (8.4)	64.55 (8.16)
Height (cm), mean (SD)	165.0 (6.5)	163.1 (7.7)	164.0 (7.1)
BMI (kg/m^2^), mean (SD)	24.0 (2.5)	24.1 (2.7)	24.1 (2.55)
18 ≤ BMI <24 (%), n (%)	9 (45.0)	9 (45.0)	18 (45.0)
24 ≤ BMI ≤28 (%), n (%)	11 (55.0)	11 (55.0)	22 (55.0)

Abbreviations: n, number; *y*, years; BMI, body mass index.

Data are presented as the mean (SD) unless otherwise noted, and categorical variables are presented as frequency (percentage).

**TABLE 2:** Baseline of two groups with reference ranges.

Variable	Reference range (unit)	Fasted (n = 20)	Fed (n = 20)	*p*
WBC	(3.5–9.5) ×10^9^/L	6.15 (1.61)	6.98 (1.56)	0.11
HGB	130–175 g/L (Male)/115–150 g/L (Female)	140.05 (14.12)	145.45 (15.16)	0.25
PLT	(125–350)×10^9^/L	253.80 (52.94)	240.65 (43.61)	0.40
ALT	(9.0–50.0)U/L (Male)/(7.0–40.0)U/L (Female)	15.75 (7.64)	16.55 (6.86)	0.73
AST	(15.0–40.0)U/L (Male)/(13.0–35.0)U/L (Female)	17.10 (2.97)	18.30 (5.06)	0.37
ALP	(45.0–125.0)U/L (Male)/(50.0–135.0)U/L (Female)	58.70 (21.26)	70.65 (26.81)	0.13
GGT	(10.0–60.0) U/L (Male)/(7.0–45.0)U/L (Female)	28.70 (25.56)	21.40 (9.06)	0.24
TBIL	(0.0–26.0)µmol/L (Male)/(0.0–21.0)µmol/L (Female)	12.16 (4.09)	12.75 (4.58)	0.67
ALB	(40.0–55.0)g/L	43.09 (2.59)	43.70 (2.54)	0.46
Ccr	(57–97)µmol/L (Male)/(41–73)µmol/L (Female)	60.95 (9.09)	59.95 (12.06)	0.77
TC	(2.6–6.0)mmol/L	4.40 (0.65)	4.24 (0.70)	0.44
TG	(0.28–1.80)mmol/L	1.14 (0.63)	1.07 (0.61)	0.73

Abbreviations: WBC, white blood cell count; HGB, hemoglobin; PLT, platelet count; ALT, alanine aminotransferase; AST, aspartate aminotransferase; ALP, alkaline phosphatase; GGT, Gamma-Glutamyl Transferase; TBIL, total bilirubin; ALB, albumin; Ccr, Creatinine Clearance Rate; TC, total cholesterol; TG, Triglyceride. Data are presented as the mean (SD) unless otherwise noted.

### 3.2 Safety

No deaths or serious adverse events (SAEs) were reported during the trial. No subjects withdrew prematurely, and all enrolled subjects were included in the safety analysis. The most common TRAEs, with an incidence rate of ≥10% are listed in Table 3. A total of 39 clinical TRAEs were reported in 21 subjects (21/40, 52.5%). Most TRAEs were grade 1 or 2, except for one subject in the fed group who had diarrhea (Grade 3). All AEs had resolved by the end of the study.17 TRAEs occurred in 10 subjects (10/20; 50%) in the fasting group, while 22 TRAEs occurred in 11 subjects (11/20; 55%) in the fed condition. The incidence of TRAEs was comparable in the two groups (50% vs. 55%). The incidence of ≥Grade 2 TRAEs was higher in the fed group (30%) compared to the fasted group (5%). In the fed group, reported ≥ Grade 2 TRAEs included oral ulceration in three participants (3/20, 15%), elevated triglycerides in one participant (1/20, 5%), elevated bilirubin in one participant (1/20, 5%), furuncle in one participant (1/20, 5%), and diarrhea in one participant (1/20, 5%). In contrast, one ≥ Grade 2 TRAE (furuncle, 5%) was observed in the fasted group. QT intervals were corrected using Fridericia’s formula (QTcF). No subject had QTcF >500 ms or ΔQTcF >60 ms during the trial.

**TABLE 3:** TRAEs in two groups (incidence rate ≥10%).

Clinical abnormality	Fasted group (n = 20)	Fed group (n = 20)
Elevated blood triglycerides, n (%)	4 (20.0)	3 (15.0)
oral ulceration, n (%)	0 (0)	4 (20.0)
Hyperuricemia, n (%)	0 (0)	2 (10.0)
Diarrhea, n (%)	1 (5.0)	2 (10.0)
Thoracalgia, n (%)	0 (0)	2 (10.0)

Data are reported as *n* (%). Abbreviations: *n*, number of TRAEs; *n* (%), incidence of subjects reporting TRAEs.

### 3.3 Food effect on PK of HEC73543 tablets

**FIGURE 2:**
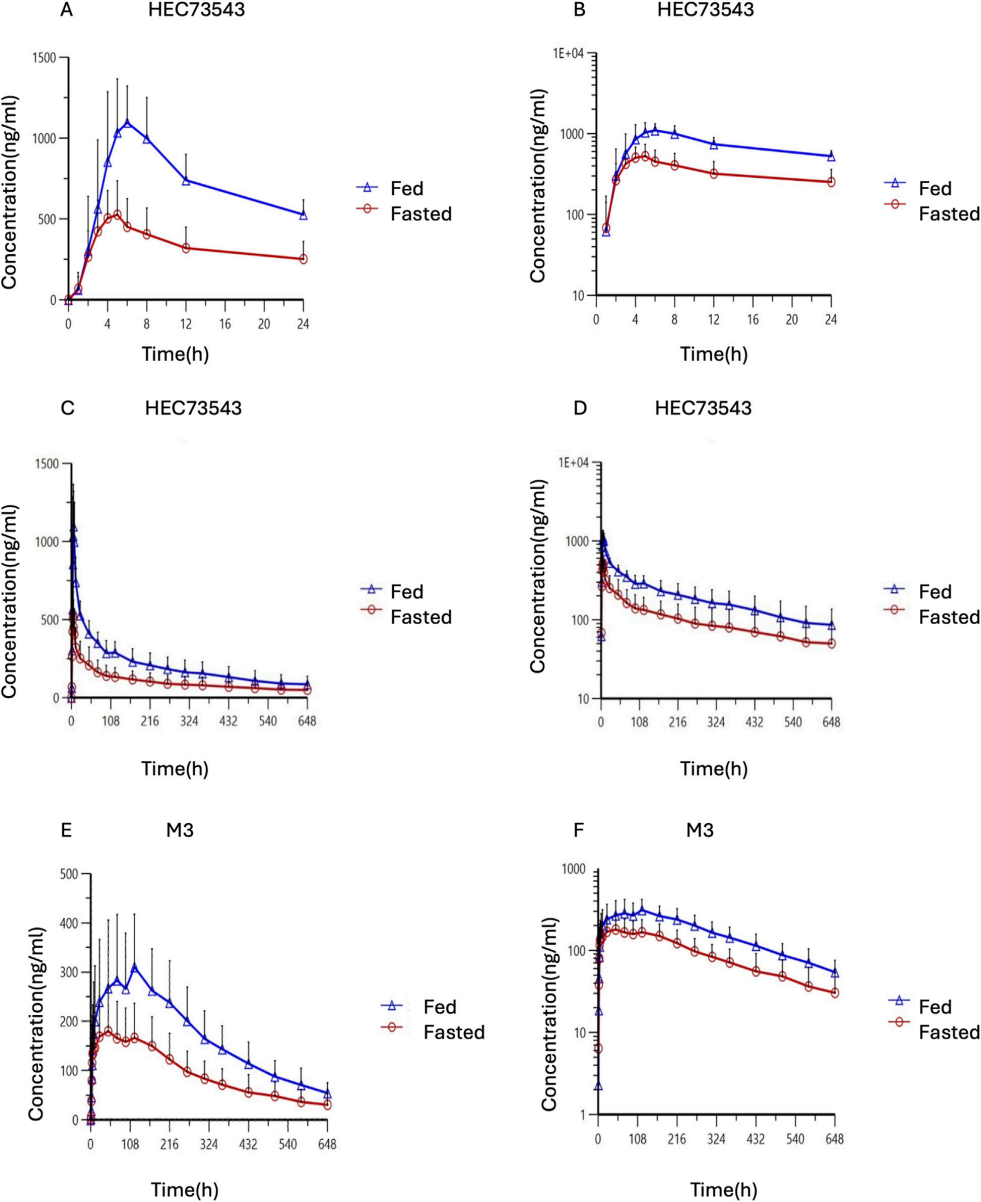
Time profiles of mean HEC73543 and M3 plasma concentration in pharmacokinetic analysis set. **(A)** Mean plasma concentration of HEC73543 measured using LC-MS/MS in fasted subjects (red, n = 20) and fed subjects (blue, n = 20) at the indicated time points (up to 24 h). **(B)** Semi-logarithmic plot of the mean plasma concentration derived from **(A)**. **(C)** Mean plasma concentration of HEC73543 measured using LC-MS/MS at the indicated time points (up to 648 h). **(D)** Semi-logarithmic plot of the mean plasma concentration derived from **(C)**. **(E)** Mean plasma concentration of M3 measured using LC-MS/MS in fasted subjects (red, n = 20) and fed subjects (blue, n = 20) at the indicated time points. **(F)** Semi-logarithmic plot of the mean plasma concentration derived from **(E)**.

The mean plasma concentration-time profiles of HEC73543 were evaluated under both fasting and fed conditions (Figure 2). The PK parameters of HEC73543 and its primary metabolite M3 are summarized in Table 4, and food effect assessment result are shown in Table 5.

**TABLE 4:** Summary of mean pharmacokinetic parameters.

Analyte	Group	t_1/2_	T_max_	C_max_	AUC_0-t_	${\text{AUC}}_{0-}\infty$	V_z_/F	CL/F	AUC_%Extrap	MRT_0-t_	${\text{MRT}}_{0-}\infty$
		(h)	(h)	(ng/mL)	(h*ng/mL)	(h*ng/mL)	(L)	(L/h)	(%)	(h)	(h)
HEC73543	Fed	258 (42.9%)	5.00 (4.00,8.00)	1,180 (19.2%)	125,779 (33.2%)	148,010 (41.0%)	101 (27.2%)	0.270 (41.0%)	16.2 (105.1%)	206 (20.5%)	367 (47.0%)
	Fasted	212 (44.3%)	4.50 (3.00,12.0)	535 (35.9%)	57,324 (56.7%)	57,994 (50.6%)	211 (43.0%)	0.690 (50.6%)	15.9 (91.3%)	205 (25.6%)	354 (55.3%)
M3	Fed	185 (26.0%)	121 (48.0,216)	312 (38.4%)	103,913 (33.5%)	118,922 (33.3%)	NA	NA	12.8 (74.2%)	241 (12.8%)	346 (31.3%)
	Fasted	189 (37.3%)	48.0 (8.00,263)	176 (60.6%)	54,445 (51.1%)	62,495 (46.8%)	NA	NA	12.3 (114.8%)	223 (19.1%)	347 (53.1%)

Data are shown as geometric mean (geometric coefficient of variation, %), except for T_max_, which is expressed as median (minimum, maximum). NA, not available. CL/F, Vz/F are apparent parameters, F (absolute bioavailability) is unknown. %AUC_extrap, percentage of extrapolated AUC.

**TABLE 5:** Food effect assessment result.

Analyte	Parameter	Geometric mean			90% CI (%)
		Fed (n = 20	Fasted (n = 20	Fed/Fasted (%)	
HEC73543	C_max_ (ng/mL)	1,180	535	221.31	190.57–257.00
	AUC_0-t_ (h*ng/mL)	125,779	57,324	219.42	173.76–277.08
	${\text{AUC}}_{0–\infty }$ (h*ng/mL)	148,010	57,994	255.22	198.10–328.80
M3	C_max_ (ng/mL)	312	176	177.48	137.80–228.58
	AUC_0-t_ (h*ng/mL)	103,913	54,445	190.86	153.26–237.68
	${\text{AUC}}_{0–\infty }$ (h*ng/mL)	118,922	62,495	190.29	151.77–238.59

The median T_max_ for HEC73543 was 5.0 h in the fed group, compared to 4.5 h in the fasted group. The 90% CIs for the GMRs of Cmax, AUC_0-t_, and ${\text{AUC}}_{0–\infty }$ of HEC73543 (fed vs. fasted conditions) were 190.57%–257.00%, 173.76%–277.08%, and 198.10%–328.80%, respectively, all of which fell outside the conventional bioequivalence range of 80.00%–125.00%. For the primary metabolite M3, the median T_max_ in the fed group was 121 h, compared to 48 h in the fasted group. Similarly, The 90% CIs for the GMRs (fed/fasted) of M3 for Cmax, AUC_0-t_, and ${\text{AUC}}_{0–\infty }$ were 137.80%–228.58%, 153.26%–237.68%, and 151.77%–238.59%, respectively, all of which fell outside the accepted bioequivalence range of 80.00%–125.00%.

## 4 Discussion

This study evaluated the PK effect of a high-fat meal on HEC73543. Compared to the fasted condition, a high-fat meal markedly enhanced the bioavailability of HEC73543, with C_max_ and ${\text{AUC}}_{0–\infty }$ increasing 1.21-fold and 1.55-fold, respectively. Similarly, M3 exhibited 0.77-fold and 0.90-fold increase in C_max_ and ${\text{AUC}}_{0–\infty }$ , following a comparable trend to the parent drug. These findings indicate that food intake increased the exposure to both the HEC73543 and its metabolites. Notably, t_1/2_ remained unchanged, indicating that the primary effect of food was to enhance absorption rather than alter drug elimination. The reduction in CL/F was primarily attributed to the increased bioavailability of HEC73543 in the fed group, rather than a decrease in intrinsic clearance. The significant increase in AUC under high-fat meal conditions can be explained by several physiological factors: 1. Increased bile salt secretion: postprandial bile salt secretion enhances the solubility of lipophilic drugs, promoting their absorption by forming bile salt-drug micelles (Charman et al., 1997). Given that HEC73543 has high lipophilicity (logP = 4.98), its solubility is likely improved in the presence of bile salts. 2. Increased splanchnic blood flow: following food intake, splanchnic blood flow increases, which may enhance oral drug transport and absorption efficiency. Enhanced hepatic blood flow may promote drug uptake from the portal circulation, as would escaping first-pass metabolism (Yan, 2017). 3. Limited impact of pH changes. Despite HEC73543 being a weak base (pKa = 8.02), this study results suggest that pH changes did not significantly affect its absorption.

In addition, we observed high variability in PK parameters, with wide 90% CIs, such as for ${\text{AUC}}_{0–\infty }$ (90% CI: 198.10–328.80%). The relatively wide CIs can be attributed to several factors. First, food effect studies involve two distinct physiological states (fed vs. fasted), and inter-subject differences in gastric pH, gastric emptying time, bile salt secretion, and intestinal motility can all impact drug absorption and metabolism. Second, the pharmacokinetic properties of HEC73543, particularly the pronounced increase in drug exposure under fed conditions, may amplify inter-individual differences in absorption and metabolism, thereby contributing to the observed variability. Third, as this is a first-in-human food-effect study with a modest sample size (n = 20 per group), it was sufficiently powered to detect a significant food effect but may not have fully accounted for inter-individual PK variability.

The impact of food on the PK profiles of FLT3 inhibitors varies. A high-fat meal slightly decreased the absorption of gilteritinib in healthy subjects, with GLSM ratios (fed/fasted %) were 74.0% for Cmax, 91.0% for AUC_72_, 93.8% for ${\text{AUC}}_{0–\infty }$ , and 94.6% for AUC_0–t_ (James et al., 2020). For midostaurin, co-administration with a standard or high-fat meal lowered C_max_ by 20%–27%, delayed T_max_ by 2.5–3 h, and increased AUC exposure by 1.2- to 1.6-fold compared to fasting condition (Kim, 2017). Quizartinib exhibited similar PK profiles under fasted and fed conditions. GLSM ratios (%) for fed/fasted were 91.58% (Cmax), 105.39% (AUC_0-t_), and 108.39% ( ${\text{AUC}}_{0–\infty }$ ) (Li et al., 2020). These differences may arise from variations in molecular structure, solubility, metabolic characteristics, and bile salt-mediated absorption mechanisms. Given the significant food effect observed with HEC73543, food intake should be considered when optimizing its administration strategy.

The safety assessments were comparable under both high-fat meal and fasted conditions. Most AEs were CTCAE Grade 1 or 2, and all adverse events resolved by the end of the study. Although the overall incidence of TRAEs was comparable between the two groups, the incidence of ≥Grade 2 TRAEs in the fed group (30%) was slightly higher than that in the fasted group (5%). Notably, three cases of Grade 2 oral ulceration were reported in the fed group. This may be mechanistically associated with FLT3 inhibition. FLT3 is a receptor tyrosine kinase expressed not only in leukemic cells but also in normal hematopoietic stem cells and mucosal epithelial progenitor cells. Inhibition of FLT3 may impair mucosal cell proliferation and repair, potentially compromising the mucosal barrier and contributing to the development of oral ulcerations. Therefore, enhanced monitoring of oral mucosal adverse events is warranted in future clinical studies, particularly under conditions associated with increased drug exposure. Although no QTcF abnormalities were observed in our food-effect study, the conclusion is constrained by the characteristics of the study population (healthy volunteers), the single-dose design, and the relatively small sample size. A comprehensive assessment of QTc liability should incorporate evidence from other clinical and nonclinical studies to determine whether a dedicated thorough QT (TQT) study is required in future development.

This study provides valuable insights into the food effect on the pharmacokinetic profile of HEC73543. However, several limitations should be acknowledged. First, the small sample size may limit the generalizability of the findings. Second, the present study was conducted exclusively in healthy Chinese adults, and HEC73543 has not yet been evaluated in clinical trials outside of China. Therefore, further studies involving diverse populations are necessary. Third, this study only evaluated a high-fat meal, while food effects may vary depending on fat content, future studies should explore moderate and low-fat meal conditions to further refine dietary recommendations.

## 5 Conclusion

Food intake significantly affects the PK profile of HEC73543, enhancing bioavailability and increasing systemic exposure to both HEC73543 and its metabolite M3.

## Data Availability

The raw data supporting the conclusions of this article will be made available by the authors, without undue reservation.
